# A Short Version of the EFECO Online Questionnaire for the Assessment of Executive Functions in School-Age Children

**DOI:** 10.3390/children8090799

**Published:** 2021-09-11

**Authors:** Sabina Barrios-Fernandez, Margarita Gozalo, Maria Amado-Fuentes, Jorge Carlos-Vivas, Andres Garcia-Gomez

**Affiliations:** 1 Social Impact and Innovation in Health (InHEALTH), University of Extremadura, 10003 Cáceres, Spain; sabinabarrios@unex.es; 2 Psychology and Anthropology Department, University of Extremadura, 10003 Cáceres, Spain; mamadofu@alumnos.unex.es; 3 Promoting a Healthy Society Research Group (PHeSO), Faculty of Sport Sciences, University of Extremadura, 10003 Cáceres, Spain; jorgecv@unex.es; 4 Education Sciences Department, University of Extremadura, 10003 Cáceres, Spain; agarcil9@unex.es

**Keywords:** executive function, children, assessment, online questionnaire

## Abstract

Executive function (EF) is a group of processes that allow individuals to be goal-oriented and to have adaptive functioning, so that adequate performance is essential for success in activities of daily living, at school and in other activities. The present study aims to create a short version of the Executive Functioning Questionnaire (EFECO) since there is a gap in the Spanish literature due to the lack of behavioural observation questionnaires at school age. A total of 3926 participants completed the online questionnaire. Subsequently, the validity and reliability of the data are analysed. The results show that the short version of the questionnaire, the EFECO-S, has a structure with five dimensions (emotional self-control, initiation, working memory, inhibition, and spatial organisation), as well as a second-order factor (global executive skill) and high reliability (ordinal Alpha = 0.68–0.88). The EFECO is composed of 67 items, while the EFECO-S has 20 items, four per factor, which turns it into a quick and easy to apply test. Therefore, it becomes an interesting alternative to be applied in screening processes with children who may be experiencing executive difficulties.

## 1. Introduction

### 1.1. Executive Function Conceptualization

Although Luria was one of the earliest authors to highlight the important role of the frontal lobe in behavioural control [1], the first definition of executive function (EF) is attributed to Lezak, who defined it as a set of cognitive abilities to carry out coherent plans for the achievement of specific goals [2]. Subsequently, EF experienced great dissemination after the publication of Phineas Gage’s story, emphasizing the role of the frontal lobe in linking cognitive, emotional and behavioural skills [3,4]. Comprehensively, EF is considered a set of cognitive and metacognitive skills that allow us to direct our behaviour towards a specific goal, including the ability to plan, carry out, check and correct our behaviours [5]. However, its conceptualization and the identification of the subprocesses or components that comprise it is a complex task due to the plurality of existing theoretical conceptualizations [6]. Thus, the term executive function is considered an umbrella concept with boundaries still undefined [7,8], with ongoing discussions about its dimensional nature, its neurological correlate and its assessment.

Although all theoretical models define EF as a set of skills, they do not all agree on the number of components nor the hierarchical structure [9,10]. The diversity of models oscillates between considering different components of the so-called cold EFs, involved in goal achievement and problem-solving, including working memory, attentional control, cognitive flexibility, inhibition, planning, organization, among others; and warm EFs, which pay attention to the role of emotional processes in cognitive control such as the ability to delay reward, self-reinforcement, decision making with affective components, empathy, theory of mind, social judgement, or emotional self-regulation [11,12]. Other authors have referred to these domains as metacognitive EF, and emotional and motivational EF [13]. Moreover, the neural network structure that serves as a substrate for executive functioning suggests that both cold and warm EF operate simultaneously in everyday life, except for dysfunctions [14]. The neural substrate for EF lies through multiple frontal-subcortical pathways among which a predominantly dorsolateral pathway is identified for cold EF processes and an orbitofrontal/ventromedial pathway for warm EF [15,16].

Furthermore, EF has a major impact on people’s daily lives both in the successful management of daily life situations and during the learning process, throughout all vital stages [8,17,18]. EF issues are related to other disorders such as mental diseases (e.g., schizophrenia, posttraumatic stress disorder) [19,20,21], and neurological disorders (e.g., Alzheimer’s disease, traumatic brain injury) [22,23]. In children, executive difficulties have been reported in neurodevelopmental disorders [24], including autism spectrum disorder [25,26,27,28], attention-deficit/hyperactivity disorder [27,29,30], intellectual disabilities [31,32,33,34], learning [35,36,37,38] and language disorders [39,40]. In addition, links have been found between deficits in EF with stress, anxiety, and depression [41]; executive difficulties have also been found in children with Gilles de LaTourette syndrome [42] and with Disruptive, Impulse-Control, and Conduct Disorders diagnosis [43], among others.

### 1.2. Executive Function Assessment

Several approaches can be considered for the EF assessment. Firstly, there are specific performance tests for evaluating specific skills such as working memory, planning, or inhibition. Thus, different tests based on the n-back, Stenberg or Stroop paradigm, go-no-go tasks, towers and maze tasks (e.g., Hanoi and London [44]), tasks with criterion shifts (e.g., Wisconsin card sorting test [45]) or fluency exist [46,47]. Examples of neuropsychological batteries which include this type of task would be the Delis Kaplan Executive Functions System (DKEFS) [48], the Minnesota Executive Function Scale (MFES) [49], and the neuropsychological assessment of executive functions in children (ENFEN) [50]. Secondly, there are non-FE specific tests such as the Wechsler scales [51] or some subscales of the Luria Neuropsychological Battery [52] which contain tasks that can be administered for EF assessment. Finally, exploratory tests through behavioural observation are also mentioned. They are usually observational scales in the form of self-report for adolescents or adults, or proxy-type scales, usually for children, to be answered by people who are close to them. Within this approach are the Behavior Rating Inventory of Executive Function (BRIEF) [53] and the Dysexecutive Questionnaire (DEX) for adults [54], and the Metacognitive Awareness System (metaCOG) [55] and the Childhood Executive Functioning Inventory (CHEXI) [56] for children.

Although the first two groups of tests (specific executive performance tasks) offer high specificity, they show weaknesses in ecological validity: they only explain 20% of the variance of EF in activities of daily living (35). Therefore, the use of specific tests in conjunction with behavioural assessment questionnaires is recommended for an adequate EF assessment [57,58]. To the best of our knowledge, there is no short version of the observational EF questionnaires available in Spanish to be used as a screening instrument.

### 1.3. The Executive Functioning Questionnaire (EFECO)

During 2011 in Spain, there was a need for a Spanish instrument to assess EF in school-age children, as the English-language tests had not yet been translated. At that time, there were some EF-specific tests or batteries, and non-specific tests to measure EF in school-age children. However, no behavioural assessment questionnaires were available for EF ecological assessment in children from 6–13 years.

The Executive Functioning Questionnaire (EFECO) creation dates from 2011, and the first version with preliminary online validation data was obtained in 2013 [59] through the Educarex platform, owned by the Regional Ministry of Education of the Government, available at https://recursos.educarex.es/cuestionarios/?cuestionarios (accessed on 1 June 2021); preliminary validation data were published in 2015 [60]; subsequently, a self-report version was developed for adolescents [61]. Currently, the EFECO has become one of the most widely used executive functioning assessment questionnaires in Spanish with more than 20,000 uses in more than 35 countries. Observations were collected on many everyday life situations in children aged between 6 and 13 years, adding reliability, and structural and construct validity data enough for reliable psychoeducational and clinical assessment.

The original EFECO version was constructed comprehensively, with a miscellany of items grouped around the most used EF subprocesses to date. The EFECO is composed of 67 items related to six factors: working memory and monitoring, inhibition, initiative and planning, organization of materials, emotional self-control, and flexibility. Responses are collected on a Likert-type scale with four response possibilities: (1) never or rarely, (2) sometimes, (3) frequently, or (4) very frequently.

### 1.4. Aim

Both the EFECO questionnaire in its proxy and self-report versions are extensive tools that require detailed reflection on multiple aspects of EF in children. It was identified the need for a screening tool for children, as there is a gap in Spanish literature to date. Between their practical implications will be the rapid detection of possible dysfunctions in EF to allow the implementation of necessary educational or clinical measures to promote the successful development of the child.

Thus, this study presents the main reliability and validity indicators of the EFECO short version, the main novelty of which is to serve as a screening tool for deviations in EF that may be influencing the daily lives of Spanish-speaking schoolchildren aged between 6–13 years.

## 2. Materials and Methods

### 2.1. Participants

The sample was obtained through more than 20,000 responses provided by families and professionals via the Education platform of the Government of Extremadura, available at https://recursos.educarex.es/cuestionarios/?cuestionarios (accessed on 1 June 2021). Eligibility criteria were: (1) respond based on the behaviours of Spanish-speaking children (2) between 6 and 13 years of age, (3) without pathology or diagnoses established by a clinical professional (4) cover the whole questionnaire, and (5) provide informed consent.

Finally, a total of 3926 children aged 6–13 years (9.15 ± 2.53) participated, being 2611 boys (66.5%), and 1315 girls (33.5%); 1945 questionnaires (49.6%) were completed by families, while 1981 (50.4%) were covered by educational or clinical professionals.

### 2.2. Instruments

The EFECO questionnaire, a behavioural assessment tool for EF assessment for Spanish-speaking children was used. As mentioned, the EFECO is composed of 67 items related to six factors: working memory and monitoring, inhibition, initiative and planning, organization of materials, emotional self-control, and flexibility. Responses are collected on a Likert-type scale with four response possibilities (1) never or rarely, (2) sometimes, (3) frequently, or (4) very frequently [59,60,61,62].

The EFECO also allows for the collection of basic socio-demographic data such as the existence or not of a clinical diagnosis, age, gender, nationality, or whether it is covered by families or professionals. The questionnaire takes between 10 and 15 min to complete. Once completed, a personalized report is generated with direct scores and percentiles.

Regarding EFECO initial psychometric indicators for the proxy version, average reliability revealed excellent values (Cronbach’s α = 0.96 and Guttman s α = 0.94). As indicators of structural validity, a factorial solution composed of 6 components explained 67.21% of the variance [60]. In the self-report for adolescents (mean age 16.23), indicators were like those from the proxy questionnaire, with internal consistency ranging from α = 0.64 to α = 0.95. Factor analysis offered a solution consisting of six grouped components with better goodness-of-fit indicators around two second-order dimensions, including a behavioural supervisory system and a cognitive supervisory system [61].

### 2.3. Procedures

As mentioned, the EFECO questionnaire is free and open to all Spanish-speaking participants, professionals, and families on the Educarex web. This platform is owned by the Regional Ministry of Education of the Government of Extremadura and is at the service of the entire universal education community in Spanish. Its main users are teachers of non-university education, school counsellors and student’s families.

Thus, data recorded during 2013–2020 for those participants who met the eligibility criteria have been processed. Then, cleaning operations were performed, deleting repeated, incomplete, or atypical questionnaires. Subsequently, a series of conceptual and statistical operations are carried out to configure the proposal for the elaboration and validation of a reduced version of the EFECO questionnaire.

This study was approved by the Bioethics and Biosafety Committee of the University of Extremadura (approval number: 70/2021). We followed the updates of the Declaration of Helsinki, modified by the 64th General Assembly of the World Medical Association (Fortaleza, Brazil, 2013) and the Law 14/2007 on Biomedical Research.

### 2.4. Statistical Analysis

The free-to-use statistical package FACTOR v.10.10.02 (Rovira I Virgili University: Tarragona, ESP) [63,64,65] was used to carry out the analyses. All of them considered the ordinal nature of the data collected through a Likert-type scale with four response options.

The Kaiser–Meyer–Olkin (KMO) and Bartlett’s sphericity tests were used as indices of sampling adequacy [66,67]. Later, to conduct the factor analysis of the items, items with factor loadings below 0.60, those with cross-loadings above 0.40 and items with communalities below 0.30 were eliminated [68]. By using a polychoric correlation matrix, suitable for ordinal data [69], the most appropriate number of dimensions was determined using optimal implementation of parallel analysis [70,71,72]. For factor extraction, the robust unweighted least squares (RULS) method with oblique rotation was used, assuming a correlation between factors [73,74]. Then, to explore the second-order factor structure, the Schmid–Leiman solution was used for the hierarchical ordering of the factors [75]. To assess the goodness-of-fit, we used the chi-squared probability setting as appropriate non-significant values (*p* > 0.05); the comparative fit index (CFI) and the non-normed fit index (NNFI); the root mean square error of approximation (RMSEA); and the root mean square of residuals (RMSR) [67,76].

Furthermore, to support construct validity, separate analyses of the questionnaires completed by families and professionals are provided. Correlations between the original version of the EFECO and the short EFECO version are reported to provide concurrent validity data. Moreover, to assess internal consistency, the Ordinal Alpha coefficient was used, considering values >0.70 as acceptable, >0.80 as good, and >0.90 excellent [77].

The statistical analysis was carried out based on secondary data from the administration of the original version of the EFECO questionnaire.

## 3. Results

### 3.1. The EFECO Short Version (EFECO-S)

**Figure 1:**
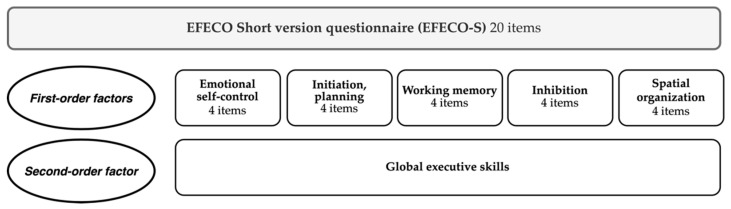
Final structure of the Executive Functioning Questionnaire (EFECO) short version.

The original version of the EFECO questionnaire is composed of 67 items. After performing the analyses, the short version (EFECO-S) was finally formed by 20 items distributed in 5 factors (Appendix A). Moreover, all first-order factors are grouped into a second-order factor (Figure 1).

This instrument is created in Spanish, but items are provided in English for easy reading. As mentioned, results are presented separately, firstly questionnaires completed by professionals, and then questionnaires answered by families.

### 3.2. Results of Questionnaires Completed by Professionals

As sample adequacy calculations offer a good fit (Bartlett’s = 22,257.4; df = 190; *p* = 0.000 and Kaiser-Meyer-Olkin test = 0.888) analyses were performed. Thus, starting with the original version of the 67-item EFECO questionnaire, and after performing the reduction procedures and factor analysis, a factorial solution of 20 items grouped into 5 dimensions was obtained.

Table 1 shows the structure and factor loadings of each item. An interpretable solution of 5 correlated dimensions was found, calling them: factor 1: Emotional self-control; factor 2: initiation and planning; factor 3: working memory; factor 4: inhibition; and factor 5: Spatial organization.

Table 2 shows the correlations between the EFECO-S factors.

Table 3 reports the EFECO-S goodness-of-fit indices from the factorial solution extracted from polychoric correlations with RULS and Robust Promin Rotation. Both model total and partial indicators show the adequate fit of the factorial model to data [68,78].

Table 4 shows the Schmid–Leiman solution used to explore the hierarchical structure of the factorial solution. It points to the existence of a unidimensional solution, grouping all first-order factors into a single second-order factor which was named global executive skill.

Table 5 shows adequate reliability indices for all the resulting dimensions through the ordinal alpha in the EFECO-S [77].

Table 6 reports the correlations between the EFECO original version (67 items) and EFECO-S (20 items), which is significant and of large magnitude.

### 3.3. Results of Questionnaires Completed by Families

Sample adequacy calculations provide a good fit: Bartlett’s = 15,221.0; *df* = 190; *p* = 0.000 and Kaiser–Meyer–Olkin test = 0.851 [67]. The factor analysis provides a factor solution composed of five correlated dimensions as in the questionnaires answered by the professionals. Table 7 shows the factor structure and loadings of every item.

Table 8 shows the correlations between the EFECO-S factors.

Table 9 shows the goodness-of-fit indices of the factorial solution. Partial and total fit statistics report an adequate fit of the factor model to the data, although data from the professionals’ questionnaires provide slightly higher indicators than those of the families.

The Schmid–Leiman solution points to a one-dimensional solution, with all first-order factors being grouped into a second-order factor (Table 10).

Adequate reliability indices for all the resulting dimensions through the ordinal alpha in the EFECO-S are found, as shown in Table 11 [77].

Finally, Table 12 illustrates that the correlation between both versions of the questionnaires answered by families is significant and of large magnitude.

## 4. Discussion

The purpose of this study was to provide a short version of the EFECO questionnaire (EFECO-S) that will provide a screening tool for EF issues to be used as screening in educational and clinical settings. Both the results provided by professionals and families offer a factorial structure with optimal indicators of goodness of fit, composed of five related dimensions that allow a global EF index (global executive skill) to be obtained. Reliability, established through ordinal alpha, is high, and the magnitude of the correlation between the original version of the EFECO questionnaire and the short version was also high, which is an important support for validity.

The five factors obtained in the short EFECO were: (1) Emotional self-control as the ability to modulate emotions to perform goal-oriented behaviours and to regulate logical, affective and emotional processes involved in decision making [79]; (2) Initiative and planning, as the ability to develop new initiatives and ideas, as well as to plan actions [80]; (3) Working memory, or the ability to maintain and manipulate information to perform tasks, solve problems or generate new information [81,82]; (4) Inhibition, defined as the ability that allows inhibiting impulses to select the most appropriate behaviour, for specific goals and adapted the social context [83]; and (5) Spatial organisation, understood as the ability to keep order in the elements to perform activities of daily living or to solve challenges [53,84].

EF are a set of cognitive and metacognitive skills that allow emotional impulses to be regulated when contrary to desired actions (EFECO-S factor 1); taking the initiative and defining goals (EFECO-S factor 2); keeping information online, maintaining online information, to monitor performance during or after the performance of activities, checking that the proposed goal has been achieved (EFECO-S factor 3); inhibiting irrelevant stimuli and others that compromise goals achievement, persevering or making changes when appropriate (EFECO-S factor 4); and maintaining an organized environment for action, including needed materials and information (EFECO-S factor 5). Thus, the EFECO-S questionnaire covers the spectrum of EF basic skills described in most of the classical approaches [2,5,53,85,86].

The second-order factor analysis did not reveal the existence of a hierarchical multifactorial structure as in other models [53]. All dimensions are correlated and clustered around a higher-order factor that was labelled as global executive skill. This hierarchical structure that gathers all dimensions around a second-order factor is in line with the results of some recent reviews that indicate that EF hierarchical structure is produced by differentiation throughout human development [87]. This evolution towards a multidimensional reality has to do with neural and functional maturation mediated by language and culture. During early childhood, inhibition and attentional control emerge unspecifically and develop in a specialised way. Between the ages of 6 and 12, skills such as goal setting and cognitive flexibility mature, and during adolescence (12–18 years), attentional control, working memory and the development of goal-setting abilities. These skills reach their peak around the age of 30, after which a slow decline in some of these skills until old age [13,80,88,89].

One of the main contributions of the present work is the presentation of a short version questionnaire, with solid indicators of reliability and validity, being an easy to use and resource-saving instrument, as it allows an EF screening of children between 6 and 13 years of age to be carried out in approximately five minutes. EFECO-S characteristics can be useful in clinical, educational and research settings. Thus, when there is a need for collective assessments either for the design of intervention programmes or for research projects, the shortness of this test will make it far more eligible than the previous version or other alternatives for EF assessment. In the context of individual intervention, the characteristics of the test (free of charge, short duration, etc.) will allow the clinical or educational professional to request its completion by parents in real time, during a session or tutoring, among others. However, the use of EFECO-S is recommended as a screening tool, and not as a diagnostic test, as diagnosis requires the use of a comprehensive assessment system including medical and neuropsychological data derived from clinical observation, medical tests, behavioural assessment questionnaires (including simulated situations in virtual reality) and performance tests [7,90,91].

This study has used an online questionnaire as the method of collecting information, as direct collection methods provide more valid results than online or telephone surveys [92,93,94]. However, online questionnaires have advantages from the researcher’s point of view, as they allow reducing costs, relocating the interviewer from the respondents, expanding the sample, and facilitating the collection and processing of data. Moreover, the data cleaning operations, together with the consistent psychometric results offered in the present work, and the consistency between the data collected by the online questionnaire and those offered in previous validation studies by the direct collection of questionnaires [60,61], are definitive support for the relevance of the sample. Furthermore, these data should be taken with caution as they are preliminary results and the analyses have been carried out based on secondary data from the application of the original version of the EFECO questionnaire. Therefore, future data should be provided, among others, about its sensitivity and specificity among different disorders, concurrent validity concerning other performance tests, and predictive validity concerning other variables. Another limitation of this short version is that it does not allow explicit assessment of some of the EF classical dimensions such as flexibility, monitoring or goal setting.

## 5. Conclusions

This study presents a short questionnaire for the EF assessment (EFECO-S) composed of five basic dimensions of executive functioning at school age. EFECO-S has reliability and validity indicators suitable both for psychoeducational and clinical purposes.

This instrument fills the existing gap in the Spanish language and can be used as screening by the educational community, including teachers and families, and health science professionals, through a simple administration that takes no more than five minutes.

## Figures and Tables

**Table 1:** EFECO short version rotated factorial solution and factor loadings.

Items	Factor 1 Emotional Self-Control	Factor 2 Initiation, Planning	Factor 3 Working Memory	Factor 4 Inhibition	Factor 5 Spatial Organization
He/she is slow in doing his home and schoolwork.			0.70		
Needs someone around of him/her to do his/her homework.			0.87		
Needs help from an adult to finish homework.			0.90		
Needs constant encouragement to start school and homework.			0.75		
Is always moving, does not standstill.				0.60	
Interferes with or disrupts the activities of others.				0.75	
Finds it difficult to behave appropriately at social gatherings.				0.77	
Can’t stop doing something when asked not to do it anymore.				0.68	
Makes decisions.		0.60			
Makes good proposals to solve problems.		0.83			
Has initiative to start activities, games, or homework.		0.64			
Has lots of ideas.		0.82			
Sometimes gets very angry about insignificant things.	0.79				
Gets very upset when he/she loses something.	0.70				
Gets upset easily.	0.84				
Has frequent mood swings (sad, happy, fearful, surprised).	0.75				
When asked to pick up his/her things, put them away neatly.					0.71
Finds materials when looking for them in his/her room or desk.					0.71
Likes to take care of his toys and belongings.					0.65
Seems to leave everything untidy in his wake.					0.63

**Table 2:** EFECO short version inter-factors correlation matrix.

	Factor 1 Emotional Self-Control	Factor 2 Initiation, Planning	Factor 3 Working Memory	Factor 4 Inhibition	Factor 5 Spatial Organization
**Factor 1** **Emotional self-control**	1				
**Factor 2** **Initiation, planning**	−0.12	1			
**Factor 3** **Working memory**	0.03	0.46	1		
**Factor 4** **Inhibition**	0.53	−0.03	0.3	1	
**Factor 5** **Spatial organization**	0.23	0.25	0.41	0.45	1

**Table 3:** EFECO-S goodness-of-fit indices from the factorial solution.

Indices	Cut-Off	Value
CMIN/DF	<2	1.87
*p* (χ2)	>0.05	0.000
NNFI	>0.90	0.996
CFI	>0.90	0.998
RMSEA	<0.06	0.021 (0.010–0.050)
RMSR	<0.08	0.0153

CMIN/DF: minimum discrepancy per degree of freedom; *p* (χ2): chi-squared probability; CFI: comparative fit index; NNFI: non-normed fit index, RMSEA: root mean square error of approximation; RMSR: root mean square of residuals.

**Table 4:** Second-order factor. Schmid–Leiman solution.

	Factor 1 Emotional Self-Control	Factor 2 Initiation, Planning	Factor 3 Working Memory	Factor 4 Inhibition	Factor 5 Spatial Organization
**Second-order factor**	0.39	0.26	0.53	0.68	0.71

**Table 5:** Internal consistency of the EFECO short version questionnaire.

	Factor 1 Emotional Self-Control	Factor 2 Initiation, Planning	Factor 3 Working Memory	Factor 4 Inhibition	Factor 5 Spatial Organization
**Ordinal Alpha**	0.85	0.81	0.88	0.79	0.77

**Table 6:** EFECO original version and short version correlations.

EFECO Original Version	Correlation (*r*)	EFECO Short Version
Emotional self-control	0.96	Emotional self-control
Initiative, planning	0.77	Initiative, planning
Working memory	0.87	Working memory
Inhibition	0.88	Inhibition
Organization	0.94	Spatial organization
Total punctuation	0.95	Total punctuation

**Table 7:** EFECO short version rotated factorial solution and factor loadings.

Items	Factor 1 Emotional Self-Control	Factor 2 Initiation, Planning	Factor 3 Working Memory	Factor 4 Inhibition	Factor 5 Spatial Organization
He/she is slow in doing his home and schoolwork.			0.70		
Needs someone around of him/her to do his/her homework.			0.75		
Needs help from an adult to finish homework.			0.84		
Needs constant encouragement to start school and homework.			0.74		
Is always moving, does not standstill.				0.59	
Interferes with or disrupts the activities of others.				0.62	
Finds it difficult to behave appropriately at social gatherings.				0.75	
Can’t stop doing something when asked not to do it anymore.				0.57	
Makes decisions.		0.62			
Makes good proposals to solve problems.		0.72			
Has initiative to start activities, games, or homework.		0.61			
Has lots of ideas.		0.81			
Sometimes gets very angry about insignificant things.	0.75				
Gets very upset when he/she loses something.	0.6				
Gets upset easily.	0.84				
Has frequent mood swings (sad, happy, fearful, surprised).	0.62				
When asked to pick up his/her things, put them away neatly.					0.59
Finds materials when looking for them in his/her room or desk.					0.55
Likes to take care of his toys and belongings.					0.6
Seems to leave everything untidy in his wake.					0.61

**Table 8:** EFECO short version inter-factors correlation matrix.

	Factor 1 Emotional Self-Control	Factor 2 Initiation, Planning	Factor 3 Working Memory	Factor 4 Inhibition	Factor 5 Spatial Organization
**Factor 1** **Emotional self-control**	1				
**Factor 2** **Initiation, planning**	−0.05	1			
**Factor 3** **Working memory**	0.25	0.13	1		
**Factor 4** **Inhibition**	0.51	−0.03	−0.1	1	
**Factor 5** **Spatial organization**	0.17	0.26	0.29	0.28	1

**Table 9:** EFECO-S goodness-of-fit indices from the factorial solution.

Indices	Cut-Off	Value
CMIN/DF	<2	2.36
*p* (χ2)	>0.05	0.000
NNFI	>0.90	0.990
CFI	>0.90	0.995
RMSEA	<0.06	0.027 (0.010–0.050)
RMSR	<0.08	0.0185

CMIN/DF: minimum discrepancy per degree of freedom; *p* (χ2): chi-squared probability; CFI: comparative fit index; NNFI: non-normed fit index, RMSEA: root mean square error of approximation; RMSR: root mean square of residuals.

**Table 10:** Second-order factor. Schmid–Leiman solution.

	Factor 1 Emotional Self-Control	Factor 2 Initiation, Planning	Factor 3 Working Memory	Factor 4 Inhibition	Factor 5 Spatial Organization
**Second-order factor**	0.58	0.05	0.49	0.78	0.40

**Table 11:** Internal consistency of the EFECO short version questionnaire.

	Factor 1 Emotional Self-Control	Factor 2 Initiation, Planning	Factor 3 Working Memory	Factor 4 Inhibition	Factor 5 Spatial Organization
**Ordinal Alpha**	0.80	0.78	0.85	0.73	0.68

**Table 12:** EFECO original version and short version correlations.

EFECO Original Version	Correlation (*r*)	EFECO Short Version
Emotional self-control	0.95	Emotional self-control
Initiative, planning	0.64	Initiative, planning
Working memory	0.84	Working memory
Inhibition	0.84	Inhibition
Organization	0.92	Spatial organization
Total punctuation	0.94	Total punctuation

## Data Availability

The datasets used during the current study are available from the corresponding author on reasonable request.

## Appendix A. Versión Breve del Cuestionario EFECO (EFECO-S)

**Autocontrol de emociones**
1. A veces se enfada mucho por cosas insignificantes.
2. Se altera mucho cuando pierde algo.
3. Se molesta fácilmente.
4. Tiene cambios de ánimo frecuentemente (triste, alegre, miedoso, sorprendido).
**Iniciativa y planificación**
5. Toma decisiones.
6. Hace propuestas buenas para resolver problemas.
7. Tiene iniciativa para comenzar actividades, juegos o tareas escolares.
8. Tiene muchas ideas.
**Memoria de trabajo**
9. Es lento en la realización de sus tareas escolares y del hogar.
10. Necesita a alguien encima para realizar sus trabajos.
11. Necesita de la ayuda de un adulto para terminar las tareas.
12. Necesita que le animen constantemente para comenzar a hacer sus tareas escolares y del hogar.
**Inhibición**
13. Está siempre moviéndose, no para quieto.
14. Interfiere o interrumpe las actividades de los demás.
15. Le resulta difícil comportarse de forma adecuada en reuniones sociales
16. Le resulta difícil dejar de hacer algo cuando se le pide que no lo haga más.
**Organización del entorno**
17. Cuando se le pide que recoja sus cosas, es capaz de recogerlas y dejarlas ordenadas.
18. Encuentra rápidamente sus materiales al buscarlos en su cuarto o escritorio.
19. Le gusta cuidar sus juguetes y sus pertenencias.
20. Parece que lo va dejando a su paso todo desordenado.
